# The effect of using blood culture bottle of bronchoalveolar larvage fluid in pneumonia

**DOI:** 10.1186/s12879-016-1591-2

**Published:** 2016-06-06

**Authors:** Eun Young Heo, Sue Shin, Hee Soon Chung, Yun-Jeong Jeong, So Hee Oh, Deog Kyeom Kim

**Affiliations:** Department of Internal Medicine, SNU-SMG Boramae Medical Center, 41, Boramaegil, Dong-jak gu, Seoul Korea; Department of Laboratory Medicine, SNU-SMG Boramae Medical Center, Seoul, Korea; Department of Internal Medicine, Dongguk University Ilsan Hospital, Dongguk University College of Medicine, Goyang, Korea; Department of Medical Statistics, SNU-SMG Boramae Medical Center, Seoul, Korea

**Keywords:** Blood culture bottle, Bronchoalveolar lavage, Pneumonia

## Abstract

**Background:**

Pneumonia is a primary cause of morbidity and mortality in infectious disease, and increasing antimicrobial resistance has raised concerns of treatment failure. Therefore, we evaluated the value of a blood culture bottle for bronchoalveolar lavage (BAL) samples on pathogen identification and on treatment modification in patients with pneumonia.

**Methods:**

We conducted a prospective study and enrolled 39 patients who were hospitalized for pneumonia. Enrolled patients underwent BAL; a 10-ml aliquot was transferred to a sterile container for standard quantitative culture, and a 5 ml aliquot was transferred to both an aerobic and an anaerobic blood culture bottle.

**Results:**

Microbes were detected in all 39 (100 %) specimens and possible pathogens were identified in 34 patients (84.6 %) from BAL blood culture bottles. In contrast, microbes were detected in 10 patients (25.6 %) and possible pathogens were isolated in 8 patients (20.5 %) in BAL fluid using conventional culture methods. Finally, 8 of 39 (20.5 %) patients changed antibiotics according to the BAL blood culture results and pneumonia improved in 6 of these patients.

**Conclusions:**

Using blood culture bottles for BAL sampling in patients with pneumonia is a sensitive method to detect pathogens in order to identify an adequate antibiotic treatment regimen.

## Background

Pneumonia is a major cause of morbidity and mortality in infectious disease [1]. Quick and effective antibiotic administration is key for successful treatment of pneumonia [2], however increasing antimicrobial resistance has raised concerns regarding the failure of pneumonia treatment. Risk and fear of resistant organisms cause physicians to overuse broad-spectrum antibiotics, possibly worsening the selection and multiplication of drug-resistant organisms. If we can isolate the causative pathogens and identify their antibiogram, particularly in hospital-acquired pneumonia (HAP)/ventilator-associated pneumonia (VAP), which is a high-risk factor for drug-resistant pathogens, physicians could more easily select adequate antibiotics and improve treatment success rates.

In order to diagnose an infection in sterile fluid such as ascites, pleural effusion, or joint fluid, use of a blood culture bottle is more sensitive than conventional culture methods to isolate pathogens [3–6]. The standard method of body fluid culture uses a solid media for culture, and before inoculating bacteria, the specimen is transferred to the laboratory and requires various procedures, such as filtration and centrifugation, to concentrate the organism. In addition, only a small amount of the specimen is inoculated on the medium lowering the probability of isolating the microorganism [7, 8]. Blood culture bottles contain enriched growth media as well as specific media to remove antibiotics, allowing for growth of microorganisms [9]. Also, inoculating body fluid specimens immediately into the medium in blood culture bottle can improve survival of the microbes.

However, unsterile body fluid, such as that from the bronchial tree, is considered unacceptable material to inoculate into blood culture bottles due to the inevitable bacterial contamination. There is no study evaluating the utility of blood culture bottles to improve the diagnostic and therapeutic significance of bronchoalveolar lavage (BAL) fluid in pneumonia. Therefore, we conducted a single-center pilot study to evaluate the effect of using a blood culture bottle with BAL fluid for pathogen identification and for treatment modification in patients with pneumonia.

## Methods

### Study subjects and design

We conducted a prospective study at Boramae Medical Center. We enrolled 39 patients who were hospitalized for pneumonia and agreed to a bronchoscopy. Pneumonia was clinically defined as the presence of a new, persistent, or progressive infiltration by chest radiograph as well as at least two of the following criteria: 1) temperature >38 °C or <35 °C; 2) leukocytosis count >10,000/mm^3^ or <3,000/mm^3^; or 3) purulent secretions. The definitions of HAP, VAP, and health care-associated pneumonia (HCAP) are summarized in Table 1.

**Table 1:** Definitions of HAP, VAP, and HCAP (7)

• HAP (hospital-acquired pneumonia)	
Pneumonia that occurs ≥48 h from time of admission	
• VAP (ventilator-associated pneumonia)	
Pneumonia that arises 48 to 72 h after endotracheal intubation	
• HCAP (healthcare-associated pneumonia)	
Prior hospitalization ≥2 days (within 90 days)	
Resided in nursing home or long-term care facility	
Received recent IV antibiotics, chemotherapy, or wound care (within 30 days)	
Attended a hospital or hemodialysis clinic	

### Sputum culture method

Sputum specimens were usually collected before antibiotic therapy was begun. The sputum collection and microbiologic evaluation were performed according to the institution’s procedure manual and a reference [10]. The patients were instructed not to expectorate saliva or postnasal discharge into the container, and were educated as how to collect expectorated sputum specimens into a sterile container. The collected sputum specimens were transferred to the microbiology laboratory for an initial Gram stain for quality evaluation based on microscopic criteria. The Gram staining results were then used to grade the specimen with the Murray and Washington’s system [11–13]. Sputum samples of grade 4 or 5 were considered acceptable and processed for further microbiological studies. Sputum cultures were processed immediately on blood agar, chocolate agar, and MacConkey agar plate.

### Bronchoscopy and culture method

A bronchoscopy and BAL were performed on enrolled patients. We performed BAL with serial 50 ml fractions of 0.9 % NaCl to a total volume of 100–150 ml using a flexible bronchoscope (Olympus MH-553, Tokyo, Japan). A 10 ml aliquot was transferred to a sterile container for standard Gram staining and quantitative culture. Pairs of aerobic and anaerobic blood culture bottles (BacT/Alert 3D System, Becton Dickinson, Sparks, Maryland, USA) were inoculated with 5 ml of retrieved BAL fluid for culture. For quantitative culture, 100 μL of BAL fluid was inoculated onto a blood agar plate, a MacConkey agar plate, a chocolate agar plate, and a Brucella blood agar plate (HANIL KOMED CO., Gyeonggi-do, Korea). Blood and MacConkey agar plates were incubated aerobically in 5 % CO_2_ at 35 °C for 18–24 h. Chocolate and Brucella blood agar plates were anaerobically incubated for up to 48 h. For the qualitative culture, 100 μl of vortexed BAL specimen was transferred to the plate; each colony from this plate was 10 colony-forming units (CFU)/ml. A colony number of more than 10^4^ CFU/ml was considered significant for a pathogen. The sediment after centrifugation of the remaining BAL fluid was used for a smear preparation for Gram staining.

The blood culture bottles containing BAL fluid were incubated in the BacT/Alert 3D System (Becton Dickinson) at 35 °C for up to 5 days. A Gram stain was performed on any positive bottles and subcultures were performed on an appropriate solid media to identify the organism and perform antibiotic susceptibility testing using a disc diffusion test.

### Analysis

The results of pathogen isolation were compared between BAL conventional culture and blood culture using BAL fluid. If one or more possible pathogenic organisms were detected from the BAL fluid in blood culture, we considered more likely pathogenic bacteria as the causative organisms. If two or more types of well-known pathogenic bacteria were isolated from the same specimen, all of them were considered pathogens of pneumonia. Gram-positive cocci without further differentiation, Gram-negative rods without differentiation, and *Candida albicans* were classified as colonizers or contamination.

This study was approved by the Institutional Review Board of Boramae Medical Center (IRB no. 06–2012–21), and was performed in accordance with the Declaration of Helsinki.

## Results

### Baseline characteristics

Bronchoscopy and BAL was performed on a total of 39 patients with pneumonia during the study period. The median age of the patients was 70.5 years and 69.2 % were male. Half of the patients (53.8 %) had community-acquired pneumonia (CAP). All except one had been treated with antibiotics before the bronchoscopy. The mean duration of the administration of antibiotics before bronchoscopy was 5.8 days (Table 2).

**Table 2:** Baseline characteristics of patients (*N* = 39)

Variables	N (%)
Age (years)	
Median (range)	70.5 (37–93)
Male	27 (69.2)
Category of pneumonia	
CAP	21 (53.8)
HCAP	6 (15.4)
HAP & VAP	12 (30.8)
Comorbidities	
Diabetes mellitus	5 (12.5)
Solid organ malignancy	11 (27.5)
Hematologic malignancy	2 (5)
Chronic respiratory disease	8 (20.5)
HIV infection	1 (2.6)
Antibiotics started on admission	38 (97.4)
Antibiotics started within 24 h before BAL	13 (33.3)
Duration of antibiotics before bronchoscopy (days), mean ± SD	5.8 ± 6.4
Leukocytes (per mm^3^), mean ± SD	11,226 ± 5056
C-reactive protein (mg/dL), mean ± SD	11.74 ± 8.24
Pneumonia outcome	
Improved	31 (77.5)
Transferred out	1 (2.5)
Died	7 (17.5)

### Comparison of culture positivity and concordance rate of isolated pathogens from BAL fluid between the conventional method and the blood culture bottle

Microbes were detected from all 39 (100 %) specimens using blood culture bottles. A single organism was isolated in 22 patients (56.4), 2 in 14 patients (35.9), and 3 or more in 3 patients (7.7 %). Except for five cases with colonizers that were unidentified Gram-positive cocci, *Delftia acidovorans*, *Pseudomonas putida*, and two *C. albicans*, possible pathogens were identified in 34 patients (84.6 %) from BAL blood culture. However, in BAL fluid conventional culture, no organisms or normal throat flora were grown in 29 patients (74.4 %), and a single microbe was detected by BAL fluid conventional culture in 10 patients (25.6 %). Except for two C. *albicans* cases, organisms that reached the qualifying > 10^4^ CFU/ml threshold were detected in 4 patients (10.3 %) among 8 patients (20.5 %), in whom possible pathogens were isolated using conventional BAL quantitative culture.

No organisms were isolated from BAL standard culture compared with BAL blood culture, in which the growth of methicillin-resistant *Staphylococcus aureus* (MRSA) and *Klebsiella* pneumonia were reported in the only patient who did not receive antibiotics before bronchoscopy. Frequency and types of isolated microorganisms are summarized in Table 3.

**Table 3:** Concordance between sputum culture, BAL quantitative culture, and BAL culture using blood culture bottles

	Sputum culture	BAL quantitative culture	BAL blood bottle culture
*Pseudomonas aeruginosa*			4 (1: ESBL)
*Klebsiella pneumoniae*	1		5 (2: ESBL)
*Staphylococcus aureus*	5 (3: MRSA)	2 (2: MRSA)	9 (7:MRSA)
Viridans streptococci			9 (1: penicillin and other beta-lactam resistant)
*Streptococcus pneumoniae*	1		1 (1: penicillin and other beta-lactam resistant)
*Acinetobacter baumannii*	2 (2: IRAB)	2 (2: IRAB)	7 (6: IRAB)
*Escherichia coli*		1	2 (2: ESBL)
*Stenotrophomonas maltophilia*		2	1
Others		MRCNS, 2 *Candida albicans*	2 *Candida albicans*, Group D Non-enterococcus, *Pseudomonas putida*, *Delftia acidovorans*

*ESBL* extended spectrum beta-lactamase, *IRAB* imipenem-resistant *Acinetobacter baumannii*, *MRSA* methicillin-resistant *Staphylococcus aureus*, *MRCNS* methicillin-resistant coagulase negative *staphylococci*

The results of pathogen isolation were consistent between BAL conventional culture and blood culture in 7 out of 10 patients who had positive results from BAL conventional culture. Among 3 patients who had discordant culture results, 2 had changed antibiotics following the BAL fluid blood culture results. In the remaining patient, MRSA through BAL conventional culture and extended spectrum beta-lactamase (ESBL) *K. pneumoniae* were isolated from BAL blood culture. The patient continued treatment with piperacillin and tazobactam with levofloxacin and the pneumonia improved. Among 7 patients who had concordant culture results, 5 changed antibiotics according to the BAL fluid culture results (1 ESBL *Escherichia coli*, 1 *Stenotrophomonas maltophilia*, and 3 imipenem-resistant *Acinetobacter baumannii* (IRAB)). The remaining 2 patients (1 *C. albicans* and 1 MRSA) continued with their antibiotics and improved.

### Impact of relying on blood culture bottle results for treatment modification

Finally, 8 of 39 (20.5 %) patients changed their antibiotics according to the BAL blood culture results, and pneumonia was improved in 6 patients (Table 4).

**Table 4:** Characteristics of patients that changed antibiotic treatments according to the BAL fluid blood culture bottle results

Patient	Pneumonia category	Conventional BAL fluid culture	BAL fluid blood culture bottle	Antibiotics before BAL	Antibiotics after BAL	Outcome
1	HAP	MRCNS	*Pseudomonas aeruginosa*	ceftazidime, vancomycin	cefepime + amikacin	improved
2	CAP	*Stenotrophomonas maltophilia*	*P. aeruginosa*	meropenem	ceftazidime, ciprofloxacin	death
3	HAP	throat normal flora	*Enterococcus faecium*, IRAB	meropenem	colistin	death
4	CAP	none	MRSA, *Klebsiella pneumoniae*	ampicillin/sulbactam	ampicillin, sulbactam, vancomycin	improved
5	CAP	none	Viridans streptococci (penicillin: I, clindamycin: R)	ceftriaxone, clindamycin	vancomycin	improved
6	HCAP	none	Viridans streptococci (penicillin: R), MRCNS	ceftriaxone, azithromycin	tazocin, levofloxacin	improved
7	HCAP	none	*K. pneumonia* (ESBL), IRAB	piperacillin + tazobactam, meropenem	colistin	improved
8	CAP	none	*S. pneumonia* (penicillin, beta lactam: R)	ceftriaxone	levofloxacin	improved

*ESBL* extended spectrum beta-lactamase, *IRAB* imipenem-resistant *Acinetobacter baumannii*, *MRSA* methicillin-resistant *Staphylococcus aureus*, *MRCNS* methicillin-resistant coagulase negative *staphylococci*

Half of the patients who changed antibiotics had CAP. In CAP, pathogens which were resistant to beta-lactam antibiotics were the main cause of delayed treatment response. In one CAP case, MRSA was isolated and pneumonia improved after adding vancomycin to the treatment regimen. In two cases of HCAP/HAP, IRABs were detected in BAL blood culture bottle samples and colistin was added to the patients’ treatment. In a 75-year-old male patient with HAP, culture results were discordant; conventional BAL fluid culture detected methicillin-resistant coagulase-negative staphylococci (MRCNS) whereas *Pseudomonas aeruginosa* was isolated in the BAL blood culture bottle. The physician adjusted the patient’s antibiotics to be more suitable for *P. aeruginosa*, which is generally more pathogenic than MRCNS, and the patient responded favorably.

Two patients died after changing antibiotics according to the BAL result from the blood culture bottle during the admission period. One 88-year-old patient had *S. maltophilia*, which was resistant to carbapenem, but sensitive to aminoglycoside, ciprofloxacin, and Bactrim, isolated in BAL fluid by conventional culture and all sensitive *P. aeruginosa* isolated in BAL fluid blood culture. Therefore, the patient’s antibiotics were stepped down from meropenem to ceftazidime and ciprofloxacin. At first, the patient improved and was moved from the intensive care unit (ICU) to the general ward. However, he could not expectorate sputum effectively and his pneumonia deteriorated again. The patient’s family rejected further invasive management and the patient died. Following his death, IRAB was isolated from the last sputum culture. The second patient had been treated for non-small cell lung cancer and died abruptly due to asphyxia. The patient’s pneumonia had been improving after changing antibiotics according to the BAL fluid culture result.

When a strategy of based on a BAL standard culture criteria with a 10^4^ CFU/ml threshold was employed, empirical antibiotic treatments were adequate in 25 out of 31 patients in whom BAL culture results were no growth or throat normal flora including *C. albicans.* However, 5 patients died in spite of adequate antibiotic therapy, whereas in 6 out of 31 patients, pneumonia was improved after adjusting antibiotics according to BAL blood culture results.

BAL standard culture results were concordant with BAL blood culture results in 3 out of 4 cases, in whom an organism was reported but did not meet the 10^4^ CFU/ml threshold in BAL standard culture; all patients changed antibiotic treatment guided by culture results. One other case (Case 1 in Table 4) with discordant results changed treatment according to the BAL blood culture results and his pneumonia improved. Among 4 patients with a number of organisms that surpassed the 10^4^ CFU/ml threshold in BAL standard culture, one patient (Case 2) changed antibiotic treatment guided by the BAL blood culture results as shown in Table 4. In the other 3 cases, initial antibiotics were inadequate and changed according to the BAL standard culture results, which were concordant with BAL blood culture results.

## Discussion

Using blood culture bottles, microorganisms were detected in all 39 patients even while being treated with antibiotics, and in 8 of these patients (20.5 %), the antibiotics were changed according to the results of the BAL fluid blood culture bottle. Though two patients died during the admission period, the change in antibiotic treatment was effective in all 8 patients.

Patients with HAP/VAP tend to undergo antibiotic treatment for a variety of reasons before the development of pneumonia. Therefore, pathogen isolation yield is generally low in patients with a high risk of harboring a drug-resistant pathogen [14, 15]. Kim et al. showed that the yield of quantitative BAL fluid culture was less than 3 % in patients who were already on antibiotics [14]. Patients with HAP or VAP, who have a high risk of multidrug-resistant pathogens, are usually being treated with antibiotics during admission, so there is less of a chance that the organism will be identified through BAL fluid quantitative culture. Therefore, using blood culture bottles can be helpful in identifying organisms present in the bronchial tree, even if they are colonizers. Routine surveillance endotracheal aspirate cultures make it possible to prescribe adequate antibiotic therapy in 95 % of patients in whom a VAP is ultimately diagnosed by BAL culture [16]. In addition, a randomized trial showed that endotracheal aspiration with non-quantitative culture of the aspirate to diagnose VAP is associated with clinical outcomes and antibiotic use similar to those associated with BAL and quantitative culture of the BAL fluid [17]. Moreover, oropharyngeal commensals may be the causative pathogens in pneumonia [18, 19].

Early, appropriate, broad-spectrum antibiotic therapy is recommended for successful HAP or VAP treatment [20]. However, this strategy carries with it the risk of selecting for drug-resistant strains. Increasing multidrug-resistant organisms are a major concern worldwide, but particularly in Asia. Chung et al. reported that the rate of multidrug-resistant (MDR) *Acinetobacter spp*. and *P. aeruginosa, which were isolated in HAP and VAP,* were 82 and 42.8 % respectively, in 10 Asian countries including Korea [21]. If we can identify the causative pathogen of a patient’s pneumonia, we can not only determine the presence of a drug-resistant pathogen and improve treatment outcome, but also provide information for de-escalating antibiotics in the case of drug-sensitive organisms. Adjusting antibiotics according to the pathogen’s antibiogram can help reduce the emergence of drug-resistant organisms that arise through misuse of broad-spectrum antibiotics.

A limitation of this study is that there is no gold standard method for isolating the exact causative pathogen in pneumonia. The bronchial tree is not a sterile space and bronchoscopy with BAL is not a sterile procedure. Therefore, the microbe isolated from the BAL fluid cannot be definitively identified as the causative pathogen of a pneumonia, particularly from a blood culture bottle in which the isolated organism cannot be quantified. However, a single organism was isolated in 22 of 39 patients, which was more than expected, from BAL fluid blood culture. Moreover, in 8 patients who were treated according to the BAL fluid blood culture results, pneumonia improved after the antibiotic treatment was adjusted.

## Conclusion

In conclusion, using blood culture bottles for BAL fluid culture in patients with pneumonia can be helpful in choosing adequate antibiotics even in patients already on antibiotic therapy. As more multidrug-resistant pathogens emerge, the importance of verifying a pathogen’s antibiogram is growing. Therefore, we need a sensitive method to isolate bacteria and using a blood culture bottle may be one way to do so in patients with pneumonia.

## Abbreviations

BAL, bronchoalveolar larvage; CAP, community-acquired pneumonia; CFU, colony-forming units; ESBL, extended spectrum beta-lactamase; HAP, hospital-acquired pneumonia; HCAP, health care-associated pneumonia; IRAB, imipenem-resistant *Acinetobacter baumannii*; MRCNS, methicillin-resistant coagulase-negative staphylococci; MRSA, methicillin-resistant *Staphylococcus aureus*; VAP, ventilator-associated pneumonia
